# Supplementary figures and images for: Peripheral alcohol metabolism dictates ethanol consumption and drinking microstructure in mice
Source: bioRxiv. 2025 Jan 13:2025.01.09.632203. Preprint. [Version 1] doi: 10.1101/2025.01.09.632203 (PMC12190315; doi:10.1101/2025.01.09.632203)

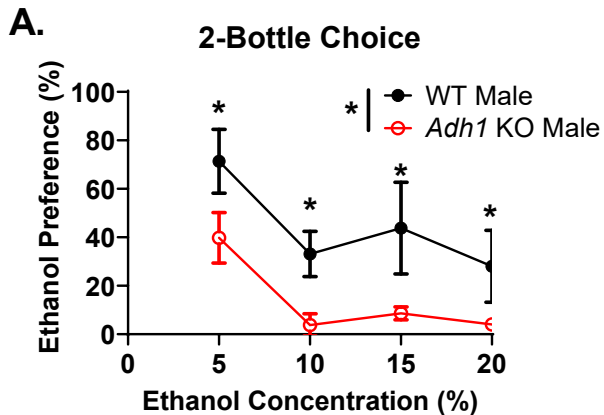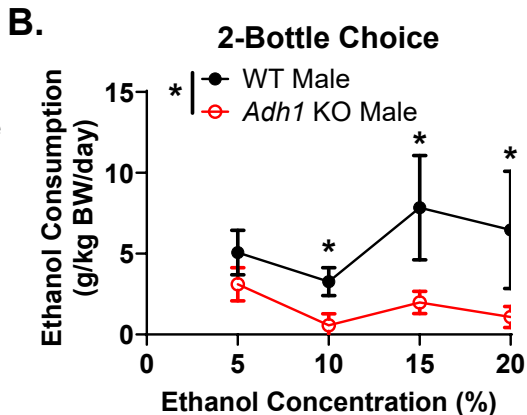

Supplement: Supplement 1 — Figure S1. Adh1 KO mice exhibit similar drinking patterns in 2BC without previous DID. Ethanol-naïve male WT and Adh1 KO mice were subjected to 2BC paradigm at escalating ethanol concentrations (5, 10, 15, 20%). [file media-1.pdf]

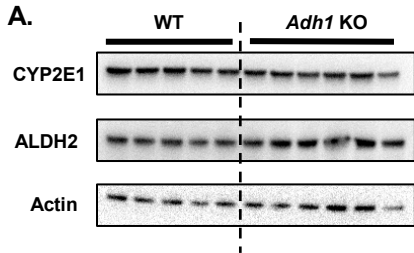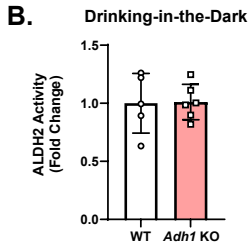

Supplement: Supplement 2 — Figure S2. Adh1 KO mice exhibit normal ALDH2 expression and activity. Protein from livers of WT and Adh1 KO mice after DID (from Fig. 4) were extracted. Western Blot detection of ALDH2 and CYP2E1 was performed with β-actin as the loading control (A). ALDH2 activity was assessed (B). [file media-2.pdf]

**A.**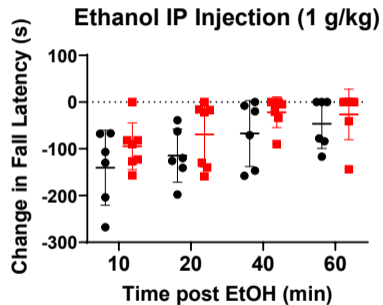**B.**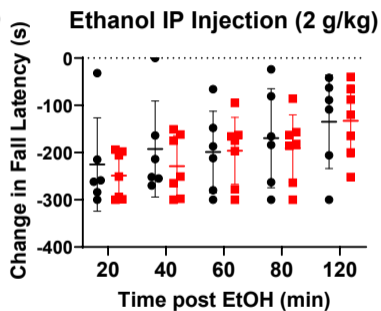**C.**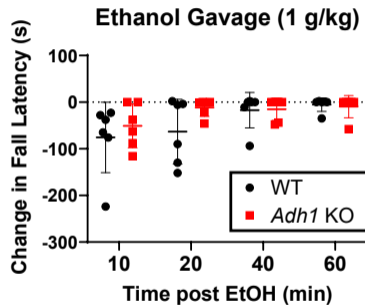

Supplement: Supplement 3 — Figure S3. Adh1 KO mice do not exhibit more impairment than WT mice after low-dose ethanol administration at early timepoints. Age-matched male WT and Adh1 KO mice were subjected to accelerating rotarod after 1 g/kg (A) or 2 g/kg (B) intraperitoneal (I.P.) ethanol injections or a 2 g/kg ethanol gavage (C). [file media-3.pdf]
